# Supplementary figures and images for: A multi-omics approach to identify the impact of miR-411ed on NSCLC TKI resistance
Source: bioRxiv. 2026 Apr 3:2026.03.31.715663. Preprint. [Version 1] doi: 10.64898/2026.03.31.715663 (PMC13060388; doi:10.64898/2026.03.31.715663)

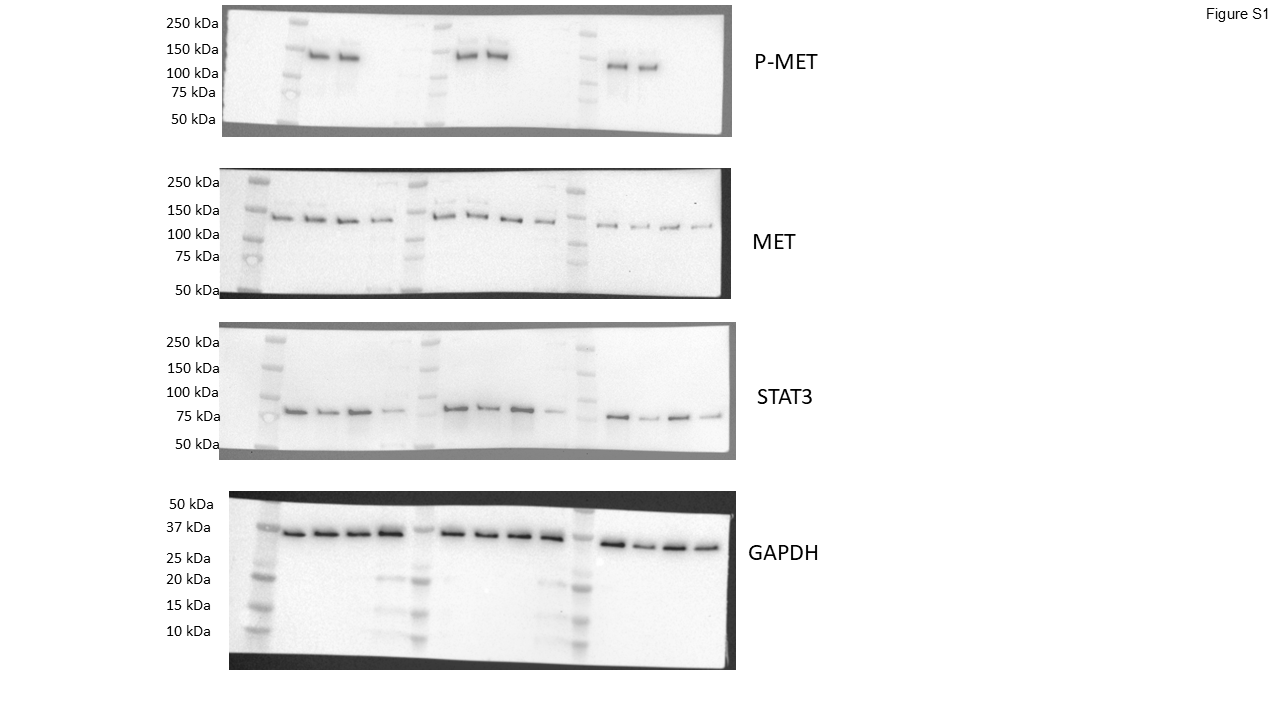

Supplement: Supplement 1 — Figure S1 Full western blot of STAT3 related to figure 1H-1I [file media-1.tif]

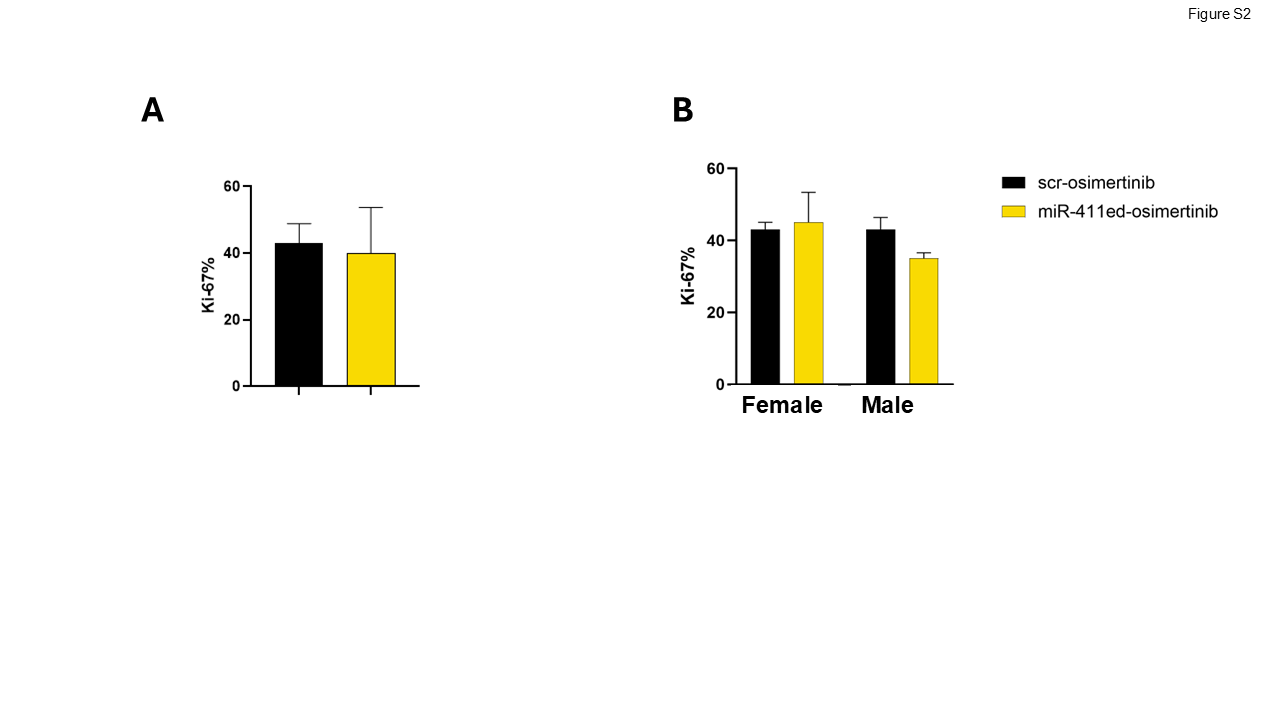

Supplement: Supplement 2 — Figure S2 Immunohistology on proliferation marker Ki-67. A) Ki-67 intensities in scr- Osimertinib and miR-411ed Osimertinib mice groups (n=10). B) Ki-67 intensities separated based on mouse sex. [file media-2.tif]
